# Street vendors in Lima in the time of COVID‐19: Guilty or oppressed?

**DOI:** 10.1111/cag.12712

**Published:** 2021-08-26

**Authors:** Diego Coletto, Lema Jaber, Linus Vanhellemont

**Affiliations:** ^1^ Department of Sociology and Social Research University of Milano‐Bicocca; ^2^ Cosmopolis Centre for Urban Research, Department of Geography Vrije Universiteit Brussel

**Keywords:** COVID‐19, informal economy, politics of difference, street vendors, Lima, COVID‐19, économie informelle, politiques de différence, vendeurs de rue, Lima

## Abstract

*The debate over the formality and informality of street vendors in Lima (Peru) is a long lasting one that has resulted in significant conflicts between the authorities and the vendors, wherein the latter are often represented as victims or people unable to comply with any type of rule. During the COVID‐19 lockdown period, the urban markets in Lima have had to abide by new safety and hygiene measures and follow new social norms to protect both vendors and buyers. This paper focuses on two food markets in the city (La Parada and Santa Anita), describing and analyzing different implementations, interpretations, and representations of the lockdown rules. The main objective is to broaden the understanding of the different types of street vendors who work in Lima and contribute to the de‐homogenization of the phenomenon. The study is based on interviews, ethnographic observations, and digital ethnography*.

## Key Messages

- For many informal workers—including street vendors—staying at home and stopping working during the pandemic cannot be considered feasible options for many reasons.
- Analyzing informal street vending activities as a homogeneous phenomenon risks provoking misleading interpretations of street vendors solely as victims or as irresponsible people.
- Recognizing there is heterogeneity in street vending helps to illuminate the different social representations, identities, and strategies used by street vendors.

## Les vendeurs de rue à Lima à l'ère de la COVID‐19: délinquants ou victimes?

*Le débat sur le statut formel ou non des vendeurs de rue à Lima au Pérou est un débat de longue date qui a donné lieu à d'importants conflits entre les autorités et les vendeurs, ces derniers étant souvent représentés soit comme des victimes, soit comme des acteurs incapables de se conformer à une quelconque règle. Pendant la période de fermeture en raison de la pandémie de COVID‐19, les marchés urbains de Lima ont dû respecter de nouvelles mesures de sécurité et d'hygiène et suivre de nouvelles normes sociales pour protéger à la fois les vendeurs et les acheteurs. Cet article se concentre sur deux marchés alimentaires de la ville (La Parada et Santa Anita), décrivant et analysant les différentes formes d'application, d'interprétation et de représentation des règles de fermeture. L'objectif principal de l'étude est d'élargir la compréhension des différents types de vendeurs de rue qui travaillent à Lima et de contribuer à l'émergence d'une vision moins réductrice de ce phénomène complexe. La démarche est basée sur des entretiens, des observations ethnographiques et une ethnographie numérique*.

## Introduction

Informal street vending plays a central role in studies on the informal economy in less economically advanced countries (Cross 2000; Forkuor et al. 2017; Recchi 2020). This form of street vending has been, and continues to be, one of the main sectors in which a large portion of the roughly 2 billion informal workers worldwide try to earn their livelihoods daily (ILO 2018).

In the literature, street vending activities are often seen as forms of pre‐modern economies or forms of free markets. Much of this literature has been dominated by a “culture of poverty” approach or, alternatively, by a neoliberalism approach. More recently, studies have begun to shed light on some important differentiations that exist among street vendors by looking at the multiplicity of ways in which street vendors build their identities and how, in the process, a politics of informality is reproduced. The notion of difference has become a means for understanding the multiple ways in which informal people practice their work and exercise their agency, and how differences are mobilized in a politics of resistance within a context of exclusionary urban governance (Crossa 2016). Specifically, this notion can refer to differences in terms of working life and working conditions (Crossa 2009). It can also refer to other aspects, such as being a migrant worker (or not), a woman or a man, a young worker (or not), or being part of an association (or not)—and, finally, it can refer to differences relative to resistance actions which allow informal people to maintain a livelihood despite structural constraints (Crossa 2009).

Drawing on this more recent approach, our objective is to broaden the understanding of the different types of street vendors who work in Lima, Peru, and contribute to the de‐homogenization of the phenomenon. We analyzed the activities of street vendors after the implementation of some restrictive measures resulting from the spread of COVID‐19 because we believe, as Arundhati Roy (2020) wrote, that lockdown situations work like a chemical experiment that suddenly illuminates hidden things.

The paper begins with a review of the literature on street vending activities in the Global South, highlighting the importance of some recent approaches that have focused attention on differences within the street vending phenomenon. We then move to the empirical study of street vendors in Lima, analyzing the main changes that have occurred in the COVID‐19 scenario. In the last section, some conclusions are advanced.

## Informal street vending and its functions in the Global South: Some insights from the literature

In the sociological literature, informal street vending has mainly been addressed for aspects concerning modernization processes, public order, and the implementation of public policies relating to urban decorum and urban renewal projects. More specifically, between the 1950s and 2000s, attention to the street vending phenomenon was continuous and consistent, especially in the Global South. Many studies increased knowledge of the informal economy and, specifically, of informal street vending, helping to identify some key features (Chen and Carré 2020). However, most of these studies provided a representation and, consequently, an analysis of informal street vending as being rather homogeneous, treating the phenomenon as a single and undifferentiated group of poor workers, who, through various strategies, survived in the urban areas of big cities in less economically advanced countries.

The first studies on street vending paid more attention to the relationship between the informal economy and modernization processes. Some interpreted informal street vending as an example of a pre‐modern economy, highlighting its function as a social safety net, which enables people to earn a livelihood when they couldn't yet enter the modern economic sectors. On the other hand, some scholars disagreed; de Soto (1989), for instance, used the example of informal street vendors to question representations that were based exclusively on the non‐economic aspects to explain their actions. He showed the costs and benefits of economic action within the informal and formal economy, concluding that, in certain ineffective institutional contexts, individuals choose to operate in the informal economy—a space where they can do business and show their individual skills; de Soto, therefore, completely redefined the informal street trade's image from one in which values and actions typical of pre‐modern and traditional societies prevailed, to a space in which individuals could act more freely from an economic point of view.

Other scholars, closer to urban studies, have instead focused attention on how large cities of the Global South have become destinations of increasing flows of migrants who, in urban contexts, have developed multiple strategies aimed at coping with unemployment and widespread poverty (Portes et al. 1989; Tockman 1991). More specifically, unplanned urbanization, economic liberalization, and privatization processes have led to growing informal economies and informal settlements. These informal activities and neighbourhoods were often ignored and marginalized by the state and municipal authorities (Thieme 2015). In this scenario, informal street markets provided easier entry‐points to the local labour market for many migrants, becoming, at the same time, an important supplier of goods and services, able to meet the growing demand of the segment of the population employed in the poorest service sectors (Sassen 2008).

### Street vendors and their differences: Observing inside the street vending phenomenon

Since the beginning of this century, an increasing number of empirical studies have paid greater attention both to the differences of various kinds of informal street vending, and to the consequences of these differences in terms of policy interventions and their effectiveness (Coletto 2013; Schindler 2014; Crossa 2016; Tucker and Anantharaman 2020). Specifically, growing attention has been paid to the so‐called “politics of informality” (Roy 2005). This perspective aims to de‐homogenize the informality of street vending activities, by looking at the multiplicity of ways in which street vendors build their identities and how, in the process, a politics and a geography of difference is reproduced (Ettlinger 2003; Crossa 2016).

Within the politics of informality approach, recent studies have analyzed the informal daily governance practices of street vendors, highlighting how the construction of the informal street vendors’ status frequently originates from the ambiguity of the formal rules governing these activities, as well as from the narratives based on the formal/informal dichotomy, which are mainly created by those who govern and administrate cities (Steel et al. 2014; Morange 2015; Adama 2020). Other studies have instead focused on informal street vending as an area in which people on the margins of the neoliberal development model can develop practices of resistance to this model, promoting forms of reciprocity, “critical” consumption, and more participatory use of public space (Hansen 2010; Graaff and Ha 2015; Berroir et al. 2016). The conflicts over the control of urban spaces, which very frequently see urban authorities and street vendors in opposing positions, have also been studied. Indeed, the dependence on public space makes street vendors particularly vulnerable because the “spaces, subjects and practices of street work” are considered a threat to “an envisaged socio‐spatial order and as a priority for intervention” (Lindell 2019, 3). Hence, the street has become a “concrete space for politics” as policymakers refashion and deploy technologies to control access, and new claims by informal people continue to materialize (Graaff and Ha 2015, 7). The analysis of bottom‐up mobilizations of street vendors has shown how daily resistance practices developed by these informal workers can take different forms, ranging from collective to individual to unplanned (Lindell 2010; Roever 2010).

More recently, the politics of informality approach was taken up and further developed by Veronica Crossa who clearly illustrated the importance of the notion of difference in understanding informality within urban spaces and the processes that define and redefine the boundaries between formal and informal. Crossa (2016) highlighted how, in the case of street vendors, it is often not only the state that actively participates in the construction of the narrative based on the dualism of formal/informal, but also the informal workers themselves who enact the formal/informal divide in contexts of displacement and exclusion. The notion of difference thus becomes crucial to understanding how the various social actors are represented and represent themselves, and how the formal/informal dichotomy is used by the social actors to define processes of social and economic inclusion and exclusion. This notion can be clearly illustrated within the context of COVID‐19 and the effects it has had on street vending activities. The full implications of the pandemic are still unclear, but it has already highlighted the changing nature and blurring boundaries between the categories of informal/formal or precarious/secure. Moreover, the pandemic has revealed that a relevant quota of essential jobs belongs to the informal economy. For this reason, the more recent literature underlines the urgency to examine labour regulations and forms of social policy that can offer protection to all types of workers—see the WIEGO (2021) study for the analysis of some effects of the pandemic on various categories of informal workers. To realize such political goals, it will be necessary to understand points of both common interest and potential conflicts between formal and informal workers, but also within the informal workers category (Cook et al. 2020).

With the approach based on the notion of difference, the focus is thus on distinctions in terms of actions, but also in terms of narratives, values, and symbols. We believe that this analytical approach can be further enriched using the concept of the social imaginary, which is rarely mentioned in the informal economy's literature. In general, various authors have referred to the social imaginary as the way in which a given group of people imagine and narrate their lives and the social reality in which they live (Castoriadis 1997). The social imaginary operates through the construction of meanings and, specifically, of myths, legends, and shared narratives. Moreover, the social imaginary is not necessarily a reflection of reality, nor is it totally the result of imagination; rather it is a set of meanings shared by a community. The construction of social imaginaries is thus closely linked to institutions, a set of shared values, and the cultural aspects that characterize a particular community, which contribute to defining how individuals perceive and understand the society to which they belong (Taylor 2004) and how they act (Benson 2012). Additionally, place appears to be a powerful stabilizing factor concerning their social practices and identities (Vanhellemont 2016). The approach of Appadurai (1996) to this concept interprets social imagination as an everyday practice that is constantly “at work.” He highlights the role that the imaginary plays in consolidating and strengthening the agency of everyone. According to Appadurai, it is the ability of individuals to imagine alternative futures that enables them to make decisions and change their lives.

The concept of the social imaginary, and specifically the interpretation given by Appadurai (1996), seems to be relevant to better understanding the differences among street vendors and the effects of the social imaginary on vendors’ agency and opportunities, highlighting some contradictions that characterize the phenomenon of street trading, where material and symbolic elements combine with different geometries.

Our paper aims to illustrate how the approach focused on differences and social imaginaries helps to explain the strategies of street vendors in Lima, who were faced with policies to reduce infection and mortality caused by the COVID‐19 pandemic. Specifically, we draw upon the experience of street vendors in La Parada and Santa Anita, two important urban markets in Lima.

## Markets and street vending activities in Lima

Many scholars have studied street vending activities in Lima, starting from the seminal work of de Soto (1989). Recently, the attention has mainly focused on the interplay between street vendors and local government, which is the main actor in defining legal‐regulatory frameworks for street vendors activities. With regard to this topic, Lima's policy orientation toward street vendors has shifted over the years—from populist support in the 1980s to policies aimed at tighter regulation in the 1990s, policies that continue into present day and are characterized by a strong neoliberal stance (Linares 2018). Despite Lima's neoliberal policy orientation, a 2014 ordinance (Ordinance No. 1787/2014) contained some pro‐poor provisions (Linares 2017). Specifically, it allowed temporary authorizations to use public space for street vending, giving preferential access to vulnerable groups in extreme poverty (Roever 2016). At the national level, the *Supreme Decree N° 005‐91‐TR* states that the street vending can be recognized as self‐employment, which occurred in context of economic crisis in response to unemployment. Moreover, with regard to the governance of street vending activities, it is important to point out that Peruvian municipalities are categorized based on their jurisdiction. There are provincial municipalities and district municipalities, and together they form the local government. Specifically, the right to regulate public space is the mandate of the provincial municipalities.

Within this legal framework, street vending activities in Lima increased over the last 20 years, fostering their central role as supply markets, especially concerning food. According to the National Census of Supply Markets, in 2016 there were 1,232 markets in Lima that include wholesale, retail, and mixed markets, with 110,229 fixed stalls, reflecting approximately the number of regular/formal vendors. The street vending scene is related to the markets because in approximately 50% of the markets, street vending activities are situated just outside them. According to Peru's household survey, in 2015 there were roughly 326,000 street vendors and around 127,000 market traders in Lima (Linares 2018).

Lima witnessed substantial growth in supply markets in the period 1970 to 2009, when more than 70% of its current markets were inaugurated, coinciding with the national migration of peasants from the countryside to the city (Mar 1986; Instituto Nacional de Estadística e Informática 2017). It is important to highlight the role of the social networks that these migrants developed based on kinship and origin ties (most of them came from the mountainous areas of Peru). These internal migrants occupied specific streets after consulting members of their social network, which led to an accumulation of vendors in areas where they shared social and cultural support (Osterling 1981).

More recently, some studies have begun to evaluate the economic effects of the COVID‐19 pandemic on formal and informal occupations in Lima. A month after the start of the pandemic, Peru witnessed for the first time a new trend in migration; people started moving from Lima back to the rural areas, reversing the trend that started in the 1950s of people immigrating from the mountainous and rural areas to Lima in search of work. Specifically, various informal street vendors, who were denied access to work due to the COVID‐19 restrictions on movement, preferred to go back to their families outside Lima. However, this is a phenomenon that for now concerns a limited number of street vendors: while 167,000 people registered to leave, only 2% (3,579) of those managed to get approvals and get transportation to leave Lima (Chavez Yacila and Turkewitz 2020).

This paper sheds light on two of the most important markets in Lima: (1) La Parada, which is known as La Parada Market, and demonstrates a vivid scene of informal street vending; and (2) the Grand Wholesale Market of Lima, also called the Santa Anita Market, which is a formal regulated indoor market, the product of a process of eviction and formalization of informal street vendors who previously worked in La Parada area. The choice of the markets is based on their main role in the city as food suppliers, and their economic relations.

### La Parada Market

**Figure 1:**
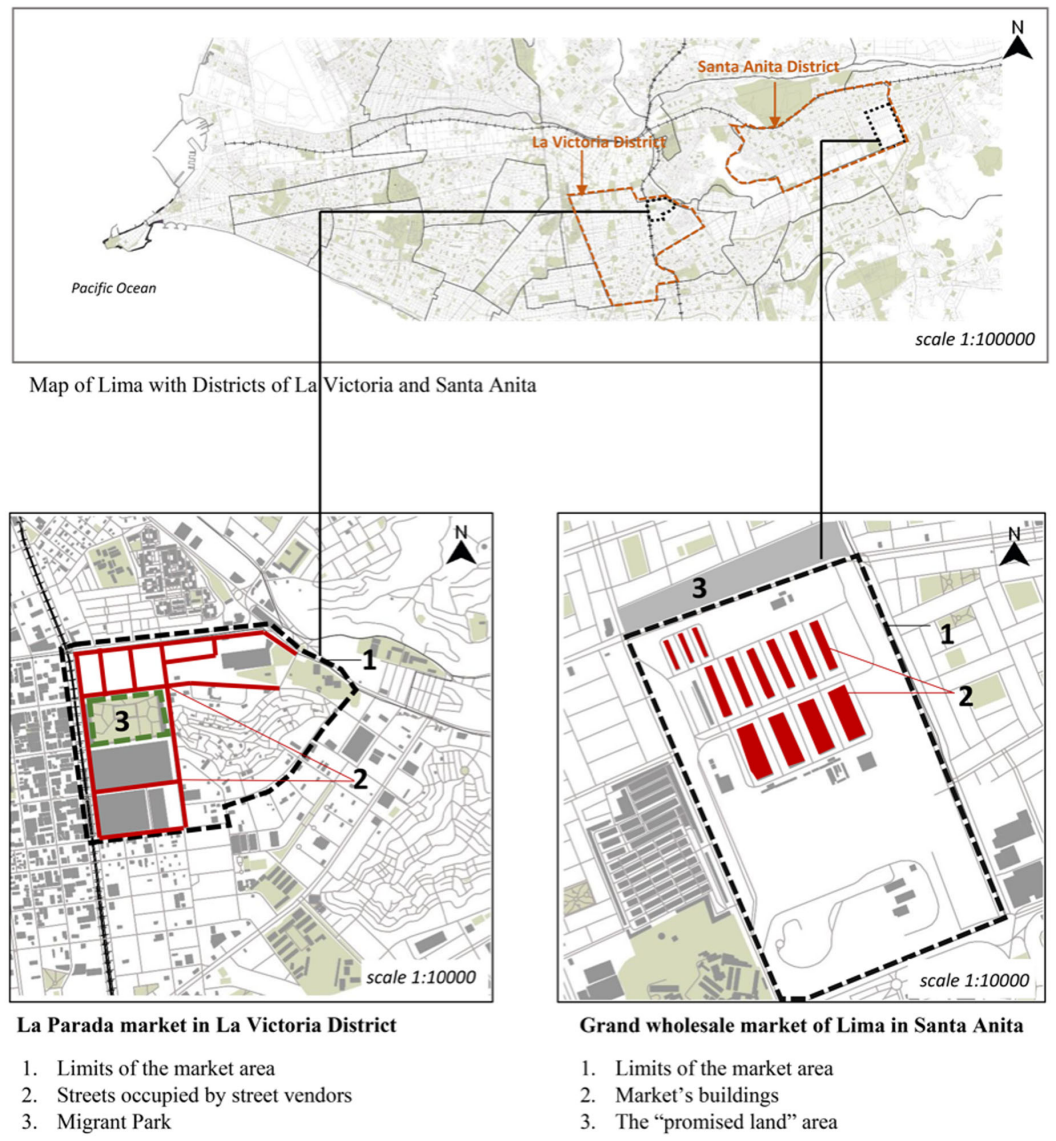
Maps of the La Parada and Santa Anita markets.SOURCE: OpenStreetMap.

The arrival of migrants in La Parada in the 1930s led to the creation and contributed to the growth of this market, due to its location. It was located at the intersection of two main highways, creating a point of departure and arrival for trucks carrying agricultural products and buses of migrants who came from the mountainous and rural areas to either earn a living in Lima or to visit family members (see Figure 1). Between 1960 and 1990, the migration process changed the face of Lima and the market started filling up rapidly. Due to the proliferation of vendors who settled in the surrounding area, public authorities began studying the possibility of relocating the market from its district (La Victoria) to another district (Santa Anita). The process of relocation went through multiple delays until 2012, when La Parada market was declared to be a public problem due to chaos, filthiness, and infrastructure deterioration. The mayor (Susana Villarán) then announced the plan to transfer it to its new location.

At the time the transfer occurred, there were more than 2,000 street vendors in the La Parada area, and the new market accepted only 640 of them, mainly due to the uncertain number and position of existing street vendors. A group of those excluded street vendors raised their claims through their associations and requested to be transferred also. They identified themselves as semi‐wholesalers because they sold perishables in large quantities and had fixed stalls at La Parada. According to their social leaders, the displacement policy was seen as an eviction; hence, they resisted the attempts to exclude them. Their demand for full relocation was received by the mayor who, after several months, announced a plan to transfer the vendors to a new wholesale market that would be built near the Santa Anita market. The new market project was called “the promised land.”

The recent pandemic has revealed the importance of relocating the street vendors. In 2020, with the COVID‐19 crisis, La Parada has emerged as a focal point of city‐wide attention (Ogando and Abizaid 2020). The TV news, for example, has focused on the daily routines of street vendors during the lockdown period. A Peru National TV headline noted that “La Parada functions like a normal day,” highlighting how roughly 8,000 street vendors worked in the streets, notwithstanding the social and economic restrictions due to the measures imposed at the national and local levels. The local population stigmatized the street vendors as being the main source of the virus, yet many of them still shopped at La Parada, especially when access was limited to other supply markets.

### Santa Anita Market

Santa Anita market is the national market of Peru that was designed and established as a substitute for La Parada market as part of the relocation plan implemented in 2012. It consists of about 1,300 stands, distributed in 16 pavilions. It is located on the Carretera Central, which is a main highway that connects Lima to the centre of Peru, passing through several provinces that are traditionally dedicated to agriculture (see Figure 1). The main objective of the creation of Santa Anita market was to modernize the trade in the city; according to the local authorities, the relocation project aimed at improving the security within the market, increasing the control of street vending activities and formalizing some of them. As mentioned above, only a portion of the vendors at La Parada were able to change their employment status to formal vendors once they were transferred to Santa Anita market. Consequently, after having been labelled as informal street vendors at La Parada, the vendors of Santa Anita market now recognize themselves as formal vendors and operate under the municipality management.

The Santa Anita market did not close during the pandemic: high control was maintained through 24‐hour security and surveillance within the market gates was strengthened. The Ministry of Health administered COVID tests to maintain the safety of both clients and vendors. Although some of the vendors tested positive, the market was not ever temporarily shut down nor labelled as being a hotspot of infection.

## Methods

This paper describes an analysis of empirical data collected from fieldwork in Lima, which lasted six months, from February to September 2020. This is the period in which the first wave of the pandemic occurred, which forced researchers to redefine the initial research project, given the impossibility of being in the field continuously. The fieldwork was thus based on both traditional qualitative research tools (ethnographic observations, interviews, document analysis) and a web‐based ethnography (Caliandro 2016). The web‐based ethnography consisted of an analysis of TV news content and online observations of Facebook street vendors’ groups in the same period.

We conducted 40 interviews in total: 8 with public administrators, 3 with representatives of the municipal company of markets; 2 with managers of the markets; 4 with neighbourhood residents; 8 with associations’ leaders; and 15 with street vendors who belong to different associations at La Parada, as well as others who work independently. The interviews had two principal objectives: (1) to clarify and explain the collected online data and documents, and (2) to collect street vendors’ narratives concerning their jobs and their perceptions of recent measures set by the authorities. Quotes from interviews are presented anonymously; the designation “PA” indicates that an interviewee was a public administrator; “AL” indicates an association leader; “NR” indicates a neighbourhood resident; and “SV” indicates a street vendor.

Other empirical material concerning street vendors was collected with the support of students in the urban planning course at the Pontificia Universidad Católica del Perú (PUCP) university. A portion of all the collected empirical data was analyzed for this paper, which can be considered a preliminary study focused on the relations between pandemic and street vending activities.

## COVID‐19 measures, working practices, and narratives

Based on the theoretical framework described above, we believe that illustrating the differences in the responses of street vendors to the COVID‐19 measures enforced in the La Parada and Santa Anita markets and describing the main narratives about the informal street vending activities can help to clarify how these daily economic activities work in Lima, and demonstrate how this street vending scenario is less homogeneous than is reported by the media.

Nationally, March 16, 2020 marked the first day of lockdown in Peru. After a few days, urban markets began to be represented by the media as hubs of infection. In a statement made by the President of Peru, he declared that the markets were the main sources of contagion (Horton 2020), and, consequently, street vending was prohibited in all areas surrounding the markets. The main objective of these measures was the dedensification of the markets; hence, they were mandated to a maximum capacity of 50%, a minimum social distance of one meter, a clear route of entry and exit, and the mandatory use of masks. These directives were mainly applied to the regular vendors in formal markets; it was more difficult to enforce them for street vendors and informal markets such as La Parada.

The design of La Parada did not facilitate the implementation of the aforementioned rules: for example, it had no allocated entrances and exits where street vendors are located. Moreover, there was no control over the market premises, and it was difficult to impose queues, particularly with the influx of people from the surrounding informal settlements and the lack of police presence. On the other hand, the Santa Anita market was deploying the social measures more strictly, made possible by its infrastructure that allowed for control over the number of vehicles entering as well as the ability to organize buyers in queues. The Santa Anita gates were controlled by a private security company, which also limited the presence of informal street vendors. Workers were given accreditation to enter the market and the public were allocated to one specific gate. The design of the market, with its separate buildings for each group of products, helped in controlling the flows of customers and workers. In addition, COVID‐19 tests were administered to vendors.

Notwithstanding the measures that limited street vending and market activities during the lockdown period—when most parts of the big supermarkets in Lima were closed—the role of urban markets as suppliers of food and other essential goods increased. At La Parada, for instance, street vendors continued to earn a living by supplying food to the city residents. Supply trucks arrived at night and business commenced on the streets at 3 am, with workers wearing their masks, gloves, hats, and uniforms.

In the La Parada area, there are around 8,000 street vendors distributed over 60 associations, which had varying responses to the new measures imposed. Many of them provided masks with their own funds. Some carried out cleaning campaigns to disinfect and fumigate the market area on a weekly basis to compensate for the difficulty in providing full social distance between vendors’ stalls. Other associations proposed that their members wear a specific uniform, which differentiated them from other street vendors, especially those who did not have fixed stalls. They also continued to hold meetings, as their leaders were keen on educating them about the new situation, as an interviewed street vendor confirmed: “They inform us on this subject and encourage us to sell our products in a more orderly manner” (SV.02).

At the individual level, street vendors at La Parada also adopted different strategies to face the pandemic and the public health measures; for instance, some older street vendors chose to stay at home temporarily and sell different homemade products online, mainly through Facebook groups. They expressed the view that their quality of life was better at home, but also not as profitable since they were making less than what they earned on the street. There were other street vendors who, in the early stages of the pandemic, abided by the COVID‐19 rules and stayed home; however, after several days of lost earnings, they were forced to return to work on the street, essentially to provide for the survival of their family.

The Santa Anita market maintained its operations, as it is a main supply for Lima and the vendors at La Parada. In general, the vendors in Santa Anita continued their vending activities, while adapting their stalls to the new sanitary requirements, committing to social distance, and wearing masks. Specifically, the vendors followed new rules concerning their sales; for example, they were asked to sell a maximum of 5 kilos to control capacity (maximum of 300 people for each pavilion). The management of the market provided masks for workers when needed, installed water taps and liquid soap, and painted the floor to demarcate safe social distancing. Clients were also monitored through temperature screening, and vulnerable people were prohibited from entering. Footbaths were installed at the pavilion doors to minimize contamination. Due to these restrictions, access to the market was denied to many informal workers, who usually went to Santa Anita to search for daily jobs such as dockworkers or cart pushers; many of them are migrants from Venezuela, who in the most recent years have left their country due to the worsening economic conditions. For this reason, in May 2020, they organized themselves in a group and crashed into the market at night trying to knock out the gate of the Santa Anita market in an act of violence.

Furthermore, during the first month of the pandemic, a curfew restricted movement between 8 pm and 5 am. This influenced the sales activity of the street vendors who went to buy their merchandise at the Santa Anita market. Previously, they would buy their goods at 1 am and reach half of their daily sales by 8 am. The limited access to the market slowed the process, restricted the vendors’ sales period, and, in general, reduced their revenues.

The pandemic brought the public administration to an impasse over two issues: (1) the need to feed the city, mainly with the markets; and (2) the threat of high virus transmission that occurred there. Specifically, the Mayor of La Victoria, George Forsythe, made the first proclamation, after he received strong pressure from the neighbours of La Parada, who complained, mainly in their Facebook groups, about the chaos of the street vendors. He therefore proposed a temporary solution to limit mass contagion by relocating the street vendors of La Parada in the so‐called Migrant Park, originally named the José María Arguedas park. The Migrant Park is the ex‐wholesale market that was relocated in Santa Anita in 2012. The Mayor of Lima, Jorge Muñoz, declined the request and proposed instead that the street vendors be moved to a nearby military barracks. The move to the barracks did not occur, and the local authorities continued to seek alternatives. They suggested moving vendors to the so‐called “promised land,” which is an area adjacent to the Santa Anita market, but the Mayor of Santa Anita, in turn, rejected that option.

Both the proposed solutions of relocation to Migrant Park and to the “promised land” have brought out resistance. As a result, a member of the Business Development Unit of the municipality suggested an integrated solution, whereby some of the La Parada vendors could be transferred to the “promised land” and another group relocated to Migrant Park, with the goal of avoiding immediate pressure in the Santa Anita district during the pandemic and facilitating a greater distance between stalls (PA.05). Beyond the proposed solutions, the pandemic helped to shed light on the street vendors’ situation at La Parada, putting them at the centre of the public debate after a long period of invisibility.

### Narratives, social representations, and positions on street vending activities during the pandemic

At the beginning of the pandemic period, the mainstream media presented La Victoria district as the hub of infection; specifically, they produced lists of the top dangerous zones and for the first few months, La Victoria appeared as a red zone and was described as “La Wuhan de Lima” (Cervantes 2020). TV reporters described the continuation of the market as an area in which “foolishness, irresponsibility and ignorance” prevailed. According to the TV reporters, this situation created fear among the neighbours of La Parada. One of the interviewed neighbours said that the areas of the district were under regular disinfection rounds with the exclusion of La Parada. The neighbourhood residents of the area expressed their resentment in Facebook groups about the lack of care and order that the La Parada zone received, and they vigorously requested that the municipality intervene. Another neighbourhood resident stated that she would have preferred more public services and police officer presence: “That day my neighbours started yelling from their window asking the authorities to enter our area for disinfection, and, finally after many calls, they did” (NR.04).

Unlike with La Parada, the media celebrated the strict monitoring efforts at the Santa Anita market and the extensive involvement of its management. TV reporters at Santa Anita highlighted the discipline of both vendors and customers at this market as well as the support of the Municipal Company of Markets, which conducted comprehensive disinfection and fumigation on their behalf on a weekly basis at all pavilions.

While the municipality of Lima was looking into solutions for La Parada, the street vendors remained in the streets and continued with their activities. The Mayor of La Victoria, however, was faced with pressure from the local population and ordered the police to seize the vendors’ products. “We are invisible to them” is the common phrase that some street vendors of La Parada often repeated. The stigma attached to all street vendors as being the “hub of infection” motivated some of them to demand relocation to the “promised land.” More specifically, this project was mainly supported by some of the young street vendors, who had emerged with new ideas and attitudes concerning their job and the formalization process, unlike the older vendors who had arrived from the mountainous areas at La Parada many years ago. Basically, some young vendors consider their products to be as important as those sold at Santa Anita; therefore, they tried to reflect the same image as the vendors at Santa Anita by wearing uniforms to indicate their compliance with the protocols, for instance.

Moreover, in the interviews and on social media, these younger vendors often highlighted the strict relations among many street vendors who work at La Parada and the wholesalers of Santa Anita. One street vendor said: “We are the followers of the wholesalers, they can't work well without us, and we can't work well without them” (SV.08). The demand for relocation to the “promised land” is perceived by these vendors as a crucial step to obtaining the same rights as their colleagues at Santa Anita; it is estimated that 32 associations, with around 1,700 street vendors, are interested in this relocation project. According to these vendors, this specific relocation plan should be part of a wider formalization process. For many of them, the formalization process would mean having a real opportunity to become a self‐employed worker; speaking of this, a street vendors’ association leader said: “Since we are self‐employed, we don't have a boss who pays our salary […] It is needed to fulfill the council agreement to go to Santa Anita because we are capable people who can work and pay […] We are not ignorant” (AL.05).

The fieldwork showed that some young street vendors of La Parada in particular seemed to be more determined than ever to solve their matter, reporting: “We are fighting to go to the promised land, the project they are doing is here on the corner, and whether it is the project or the pandemic, we are going to end up dying, so we have to withdraw” (SV.03). For these vendors, the situation at La Parada is no longer sustainable and, consequently, they tried to “use” the pandemic situation to become recognized actors in the public debate concerning the relocation project.

There are, however, different positions among street vendors concerning the relocation project and the future of street vending activities in the La Parada area. Some senior street vendors, for instance, were not interested in the relocation. They expressed a preference for staying in La Parada with statements like “They say that in Santa Anita there is nothing, we have to be here […] In the promised land, all the people will be registered, and we have nothing to gain with this action; it's not useful to go and people in Santa Anita don't want us there” (SV.13).

Different collective and individual actions, narratives, and social representations suggest that relevant differences are present within the street vendors’ category in terms of motivations and perspectives, and in terms of meanings conferred to the street vending activities and formal/informal boundaries. These are differences that can lead to the presumption of the presence of different identities among the street vendors, which are based on different values and beliefs. But more time in the field is needed to better investigate and explain these aspects.

## Conclusions

The COVID‐19 pandemic has exacerbated some old inequalities and created new ones among informal workers. The economic crisis is expected to increase the poverty rates and expand extreme poverty, with the risk that informal workers will be among those most affected by these trends. Specifically, ILO (2020) warns that 1.6 billion informal workers are at risk of losing their livelihoods during the pandemic.

In this context, this paper has highlighted that the hashtag #IStayHome does not have the same meaning for everyone; for many informal workers—including street vendors—staying at home and stopping working cannot be considered a feasible option for a variety of reasons. Moreover, the pandemic seems to have redefined the movement between “visibility” and “invisibility” that characterizes many informal activities. In the case of street vendors, they obtained greater visibility in the last months. In part, this is because they have been identified as subjects at both personal and community risk from a health point of view; in part it is also because their crucial role in the urban economy, guaranteeing the supply of basic goods and services to a significant portion of the population during difficult times, has become evident.

Analyzing informal street vending activities as a homogeneous phenomenon risks provoking misleading interpretations of street vendors solely as either victims or as irresponsible people who fail to comply with COVID‐19 measures, endangering public health. The La Parada and Santa Anita cases shed light on important differences in this sense. Vending activities did not cease in the two areas, highlighting the vital role of popular, urban markets during the pandemic. At the same time, the prevalent working and health conditions in the two zones were very different. An effective private‐public cooperation in the Santa Anita market has allowed activities to continue safely, with clear communication to the public that the activities were being controlled. At the same time, in this context, the effective enforcement of the last COVID measures provoked a further marginalization of the most precarious informal workers; among them there is a significant share of Venezuelan migrants without any residence permit. In the La Parada area, the lack of state involvement was more evident, as well as the perception of a widespread disorder. But the dichotomy of formal (Santa Anita)/informal (La Parada) is not enough to understand street vending activities and their agency in these urban areas.

Indeed, in the La Parada market forms of self‐regulation were created from the bottom up, making the market very fragmented with regard to approaches to work activities in difficult health conditions. The need for survival prevailed, which prompted some street vendors to continue their work without taking any precautions. On the other hand, some street vendors instead adopted strategies aimed at reconciling work and safety, even in a scenario where these two terms have been represented by the mainstream media as irreconcilable.

We have shown that the description and analysis of narratives and social imaginaries concerning street vending activities can be crucial to aid in highlighting and understanding differences within the street vendors’ category. Specifically, some common narratives contributed to strengthen the agency of specific groups of street vendors. In this analysis, we mainly reference associations of young vendors that have attempted, through material and symbolic actions, to build a different perception of informal street vendors, with the aim of bettering their current situation. Attempts to bring awareness to the reality of some street vendors at La Parada closer to that of the sellers of Santa Anita are, in fact, aimed at changing the future for some of them by promoting the implementation of the new relocation project. These narratives suggest that different meanings are attributed to the street vending activities, which are used to strengthen the cohesion within specific groups of street vendors, legitimizing their “voice” in the difficult dialogue with public authorities. At the same time, they represent everyday practices that become social imaginaries able to affect decisions and imagine different futures. Hence, the street becomes a space for politics (Graaff and Ha 2015), as new and different claims by different groups of street vendors continue to materialize and, contemporaneously, local authorities redefine uses and access to the urban areas.

In general, the differences outlined in this paper—which mainly refer to actions, narratives, and social imaginaries—seem to suggest attempts are being made to build different identities for street vendors. The presence of these differences signals the existence of different motivations, values, meanings, and needs that could require diversified interventions. In this sense, being an informal street vendor and also an internal or international migrant is a condition that can create further differences in terms of shared identities. For instance, the fieldwork showed some important differences between age differences of street vendors, many of whom arrived in Lima from the mountainous areas of the country in different periods of the last 30 years. This is an aspect that appeared only marginally in this first exploratory study, but which will certainly need to be deepened; in fact, it would be a useful contribution for the literature, as the disentanglement of various situations of migration, at various temporal and spatial scales (both national and international), that are at play in the relationship of street vendors with urban societies still needs to be addressed.

In general, the process of de‐homogenization of the street vending activities can be useful for improving the understanding of the phenomenon, both in terms of highlighting different positions in everyday street vending practices and how these differences can be mobilized in the public debate, and in increasing knowledge and competencies for policy makers and urban planners tasked with solving critical issues related to this type of informality. For these reasons, further empirical studies and analysis are needed.
